# Delayed bleeding due to a sharp protruding edge of the endostaplers after a wedge resection of the lung: a case report

**DOI:** 10.1186/s40792-020-0797-0

**Published:** 2020-01-30

**Authors:** Kazuki Yamano, Ryo Fujikawa, Toru Nakamura

**Affiliations:** Department of General Thoracic Surgery, Seirei Hamamatsu General Hospital, 2-12-12 Sumiyoshi, Hamamatsu, 430-8558 Japan

**Keywords:** Postoperative bleeding, Video-assisted thoracoscopic surgery, Endostapler

## Abstract

**Background:**

Endoscopic devices often cause device-related surgical morbidities such as postoperative bleeding. Delayed bleeding due to a protruding edge of an endostapler has not been previously described in the literature.

**Case presentation:**

An 80-yr-old man with a second primary lung cancer underwent a wedge resection of the right lower lobe. He developed sudden hypotension and massive bleeding from the chest tube 4 h after the surgery and underwent an emergency reoperation. A torn parietal pleura was found to have caused a persistent bleeding. There was a sharp protruding edge created by multiple firings of the endostapler. The subsequent lung expansion would have promoted a direct contact between the edge and parietal pleura resulting in delayed bleeding.

**Conclusions:**

A protruding edge due to multiple firings of an endostapler could injure the parietal pleura and cause delayed bleeding after a lung resection. This type of injury would be more common in wedge resection cases because of the larger residual lung volume preserved, which is expected to have a better lung expansion and facilitate the direct contact of the staple line and parietal pleura.

## Background

Endoscopic devices play an important role in a safe video-assisted thoracoscopic surgery (VATS). Furthermore, they often cause device-related surgical morbidities such as postoperative bleeding. We herein report a case of delayed bleeding caused by a sharp protruding edge of the endostaplers, which required an emergency reoperation.

## Case presentation

**Fig. 1:**
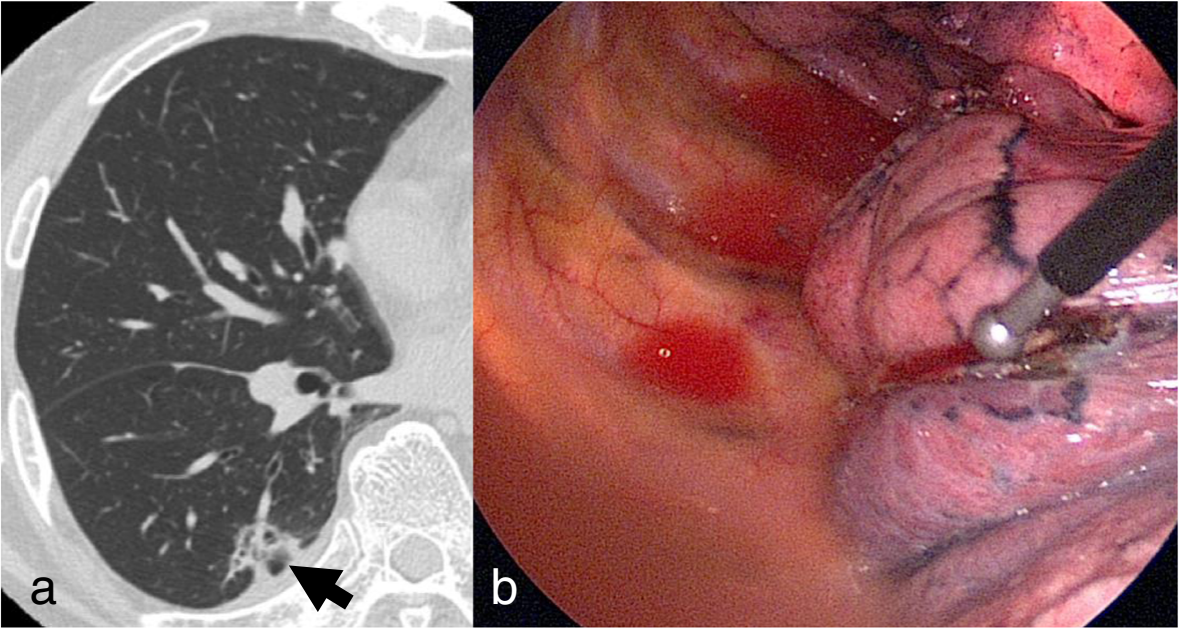
**a** Chest computed tomography showing an ill-defined nodule in the right lower lobe (arrow). **b** There was no bleeding from the parietal pleura immediately before the end of the first surgery

**Fig. 2:**
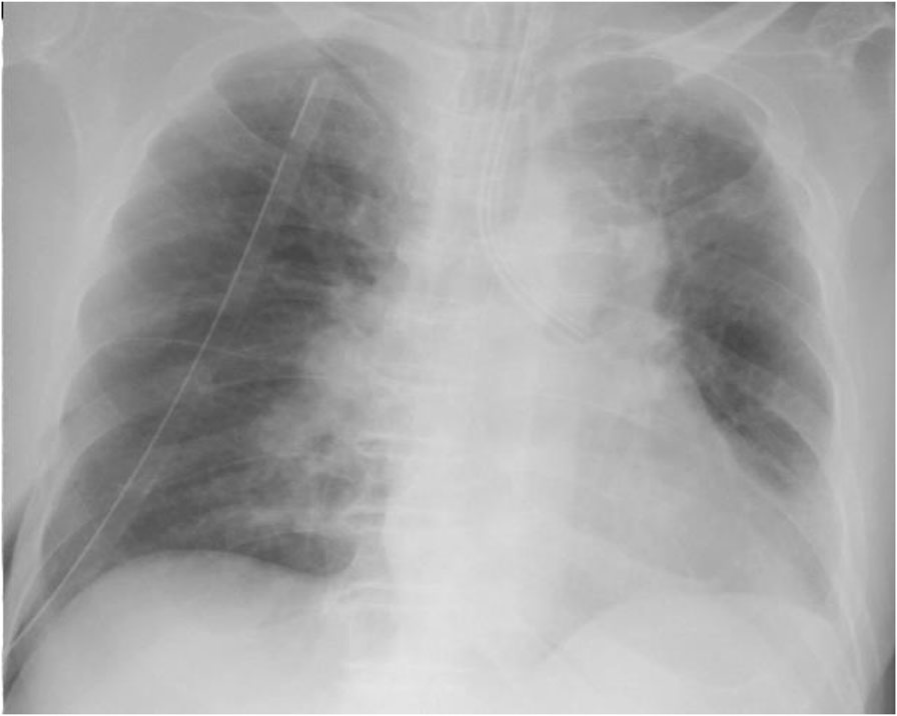
Chest radiograph after the wedge resection of the lung showing excellent lung expansion

An 80-yr-old man presented with a lung nodule in the right lower lobe detected by chest computed tomography (CT) (Fig. 1a). He had undergone a left upper lung lobectomy for a pathological T3N0M0 Stage II B primary adenocarcinoma 7 yr prior. Transbronchial biopsy revealed a poorly differentiated non-small-cell lung cancer and a further radiological examination revealed no metastatic lesions. He was diagnosed with clinical T1bN0M0 Stage I second primary lung cancer and underwent a VATS wedge resection using an endostapler (Powered Echelon45®; Ethicon Endo-Surgery) four times (three gold and one green cartridge) without any air leakage or bleeding (Fig. 1b). No fibrin glue or sealant was used. The operative time was 60 min and the postoperative lung expansion was satisfactory (Fig. 2).

**Fig. 3:**
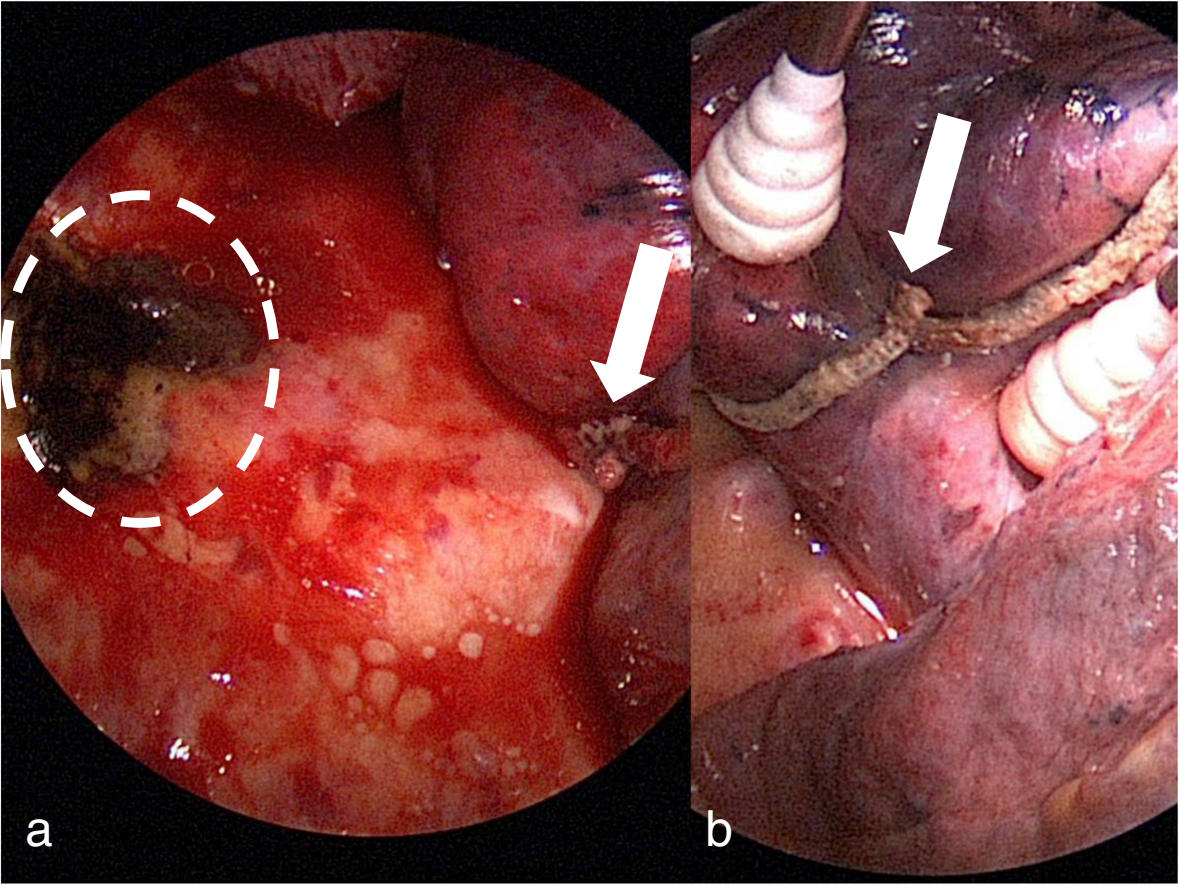
A surgical view of the reoperation. **a** Hemostasis of the persistent bleeding from the parietal pleura (dot line) was achieved by electrocautery ablation. **b** The sharp protruding edge of the staple line (arrow) would have injured the parietal pleura

He developed a sudden hypotension with a blood pressure of 70/40 mmHg and a massive bleeding from the chest tube 4 h after the surgery and underwent an emergency reoperation. After evacuating a blood clot, the dorsal chest wall was found to have caused a persistent bleeding from the torn parietal pleura. Prompt hemostasis was achieved by electrocautery ablation and there were no other bleeding sites. Further exploration revealed a sharp protruding edge due to the intersection of the endostaplers (Fig. 3a). Those findings suggested that this protruding edge had injured the corresponding parietal pleura by lung expansion (Fig. 3b). We overlaid the injured parietal pleura with oxidized regenerated cellulose and the edge of the staple line with polyglycolic acid, respectively. The total amount of bleeding was 1330 g. The post re-operative course was uneventful.

## Discussion

Postoperative bleeding requiring a reoperation after a VATS procedure is reported to be 0.6% [1] and surgical devices are often responsible [2–5]. An endostapler is one of the most essential devices for a VATS and could cause parenchymal injury by the direct contact of the reinforced materials [3, 6] or tip of the stapler itself [2, 5]. However, the delayed bleeding due to the protruding edge of the staple line has not been previously described in the literature.

In our case, multiple firings of the endostapler created a sharp protruding edge, and the subsequent lung expansion would have promoted a direct contact between the protruding edge and parietal pleura resulting in delayed bleeding. This risk would be more significant in wedge resection cases because of the larger residual lung volume preserved which is expected to have a better lung expansion and facilitate the direct contact of the staple line and parietal pleura.

We should have paid much more attention to prevent an irregular cut line by adjusting the trajectory and insertion angle of the endostapler. Further, we should have overlaid the staple line with a surgical sealant once the protruding edge became evident. The physical isolation between the lung and parietal pleura might have avoided the contact bleeding.

## Conclusions

A protruding edge by the multiple firings of endostaplers could injure the parietal pleura and cause delayed bleeding after lung resection. This type of injury would be more common in wedge resection cases because of the larger lung volume preserved than in segmentectomy or lobectomy cases. Once the protruding edge becomes evident during surgery, it should be overlaid with surgical sealant to avoid contact bleeding.

## Data Availability

Not applicable.

## Abbreviations

CT: Computed tomography
VATS: Video-assisted thoracoscopic surgery
